# Tlr7 drives sex- and tissue-dependent effects in Sjögren’s disease

**DOI:** 10.3389/fcell.2024.1434269

**Published:** 2024-09-06

**Authors:** Achamaporn Punnanitinont, Sheta Biswas, Eileen M. Kasperek, Jason Osinski, Chengsong Zhu, Jeffrey C. Miecznikowski, Rose-Anne Romano, Jill M. Kramer

**Affiliations:** ^1^ Department of Oral Biology, School of Dental Medicine, The University at Buffalo, State University of New York, Buffalo, NY, United States; ^2^ Department of Biochemistry and Molecular Biology, Noakhali Science and Technology University, Noakhali, Bangladesh; ^3^ Department of Immunology, Microarray and Immune Phenotyping Core Facility, University of Texas Southwestern Medical Center, Dallas, TX, United States; ^4^ Department of Biostatistics, School of Public Health and Health Professions, The University at Buffalo, State University of New York, Buffalo, NY, United States

**Keywords:** saliva, sialadenitis, age-associated B cells (ABC), NOD.B10, autoantibodies

## Abstract

Primary Sjögren’s disease (pSD) is a systemic autoimmune disease that has the strongest female predilection of all autoimmune diseases. The underlying mechanisms that govern this sexual dimorphism, however, remain poorly understood. We hypothesized that pSD females would exhibit more robust disease as compared to males, and that Tlr7 controls distinct disease manifestations in males and females. Using a well-established pSD mouse model, we harvested exocrine glands, and pulmonary and renal tissue from males and females and quantified the inflammation present. We then collected salivary glands, spleens, and cervical lymph nodes and performed flow cytometry to assess immune populations implicated in disease. We also harvested sera to examine total and autoreactive antibodies. Our data revealed that pSD mice displayed sex-biased disease, as pSD females showed decreased dacryoadenitis, but increased nephritis as compared to males. Moreover, females exhibited increased proportions of germinal center B cells and CD4^+^ activated/memory T cells in the periphery. Additionally, salivary gland immune populations were altered in a sex-dependent manner in pSD. Females with pSD also displayed elevated total and autoreactive IgG as compared to males. Additionally, splenic B cell Tlr7 expression was increased in females. We next generated pSD mice that lacked *Tlr7* systemically and found that ablation of Tlr*7* was primarily protective in pSD females, while Tlr7-deficient pSD males showed heightened disease. Thus, pSD mice display sex-biased disease and these dichotomous manifestations are governed by Tlr7 activation. This study identifies Tlr7 as a druggable target for pSD, and highlights the importance of studying pSD disease mechanisms in both sexes.

## Introduction

In the United States, autoimmune diseases are the third most common type of disease, affecting 8% of the population. Of those afflicted, 80% are women (Fairweather and Rose, 2004). Among the major autoimmune diseases, primary Sjögren’s disease (pSD, also referred to as primary Sjögren’s syndrome) displays the most striking female predilection (Jiwrajka and Anguera, 2022). Primary SD is a systemic autoimmune disorder that affects the exocrine tissues, including salivary and lacrimal glands, leading to glandular dysfunction (Mariette and Criswell, 2018). As a result, pSD patients can suffer from dry mouth and eyes with debilitating consequences that negatively impact the overall quality of life (Mariette and Criswell, 2018). In addition to exocrinopathy, extra-glandular manifestations are observed in 30%–40% of pSD patients, such as nephritis and interstitial pneumonitis (Mariette and Criswell, 2018). Notably, although females develop pSD far more commonly than males, some disease manifestations are more severe in males, as males with pSD have an increased incidence of pulmonary involvement and lymphoma as compared to female pSD patients (Park et al., 2020; Ramirez Sepulveda et al., 2017). Due to its rarity, however, the disease is understudied in males and most patient cohorts analyzed are primarily or exclusively females.

While the reasons underlying this sexual dimorphism are incompletely understood, both sex hormones and altered expression of X-linked genes contribute to autoimmunity (Thorlacius et al., 2023; Punnanitinont and Kramer, 2023). There are several immune-related genes located in the X chromosome, the altered expression of which may mediate sex-specific differences in immune function in the context of health and disease (Jiwrajka and Anguera, 2022). Typically, X-linked genes are subject to X-chromosome inactivation (XCI) in females to ensure equivalent gene dosage in both sexes (Souyris et al., 2019). X-linked genes, however, can escape XCI, resulting in overexpression in females and this has important implications in many autoimmune diseases. Indeed, several immune-related genes associated with pSD pathogenesis, such as *TLR7, TLR8, BTK* and CD40 ligand (*CD40LG),* are located on the X chromosome (Punnanitinont and Kramer, 2023). TLR7 has been extensively studied in both humans and mouse models. This receptor drives the development of Systemic Lupus Erythematosus (SLE or lupus), an autoimmune disease that shares overlapping clinical and molecular features with pSD, yet is a distinct disease (Pisitkun et al., 2006; Soni et al., 2014; Satterthwaite, 2021). Several GWAS studies revealed that *TLR7* is a risk allele associated with SLE development (dos Santos et al., 2012; Shen et al., 2010; Wang et al., 2014). Moreover, murine studies demonstrate an important role for Tlr7 in disease development, and B cell-intrinsic TLR7 plays a vital role in driving SLE in both humans and mice (Soni et al., 2014; Satterthwaite, 2021; Fillatreau et al., 2021).

Previously, our group demonstrated that Myd88-mediated signaling was required to drive local and systemic disease development in a pSD mouse model (Kiripolsky et al., 2017a; Kiripolsky et al., 2020; Kiripolsky et al., 2021). We and others have performed genetic studies that demonstrated that MyD88-dependent pathways are dysregulated in salivary tissue derived from pSD patients (Verstappen et al., 2021; Oyelakin et al., 2020). However, the specific receptors that activate MyD88-dependent signaling cascades in the context of pSD remain poorly understood. Since TLR7 is implicated in several autoimmune diseases and this receptor relies on MyD88 for signaling, we sought to examine the role of Tlr7 in pSD. Prior studies by our group have demonstrated that Tlr7 agonism accelerates disease in female pSD mice and drives expansion of age-associated B cells (ABCs), a B cell subset that mediates autoimmunity (Punnanitinont et al., 2022; Punnanitinont et al., 2024; Cancro, 2020; Phalke et al., 2022). Several murine studies further corroborate our findings, demonstrating that ABCs mediate immune dysfunction in lupus in a Tlr7-dependent manner (Phalke et al., 2022; Ricker et al., 2021; Rubtsov et al., 2011; Rubtsov et al., 2013). In addition, our previously published data revealed that Tlr7 is highly expressed in B cells, and ABC subsets were expanded both in the context of spontaneous disease progression and following Tlr7 agonism in female pSD mice (Punnanitinont et al., 2024). While these studies demonstrate that exogenous activation of Tlr7 can drive pSD, further work is needed to decipher the role of Tlr7 signals in this disease. In this current study, we hypothesized that systemic ablation of Tlr7 would ameliorate pSD development in a sex-specific manner.

We utilized a well-characterized mouse model for these studies, termed NOD.B10Sn-*H2* ^*b*^/J (NOD.B10). NOD.B10 mice recapitulate many aspects of pSD observed in patients. For example, NOD.B10 females develop pSD spontaneously by 26 weeks of age, as evident by loss of salivary flow, robust inflammation in several tissues, including the exocrine glands, lungs, and kidneys, and increased production of anti-nuclear autoantibodies (ANAs) as compared to healthy controls (Kiripolsky et al., 2017b; Robinson et al., 1998). Of note, previous studies in NOD.B10 mice have been performed almost exclusively in females, so disease progression and severity remain poorly understood in males in this model.

To examine the role of Tlr7 on disease development in females and males, we generated NOD.B10 mice that lacked systemic expression of *Tlr7* (NOD.B10^*Tlr7−/−*^ or NOD.B10^*Tlr7-/y*^). Our studies revealed that NOD.B10 mice display sex-biased disease manifestations, and overall disease severity was worse in females as compared to males at the clinical disease stage. Ablation of *Tlr7* altered disease manifestations in a sex-specific manner, and had dichotomous effects in males and females. NOD.B10^*Tlr7−/−*^ females were primarily protected from pSD, while *Tlr7*-deficient males showed exacerbated disease, indicating that Tlr7 activation has opposing roles in males and females in the context of pSD. Results from this study carry significant clinical relevance regarding design of therapeutics that target specific disease manifestations, and reveal novel sex- and tissue-specific roles for Tlr7 in pSD.

## Materials and methods

### Mice

NOD.B10Sn-*H2* ^b^ */*J (NOD.B10) mice were acquired from Jackson Laboratories (stock #002591). To generate *Tlr7*-deficient animals on the NOD.B10 genetic background, B6.129S1-*Tlr7* ^*tm1Flv*^/J mice (stock #008380) were bred to the NOD.B10 strain for 7 generations. The resultant animals were verified to be congenic with the NOD.B10 strain using a speed congenics approach (Jackson Laboratories). All animals used in this study were at least 6 and 7 months of age at the time of euthanasia, which represents the clinical disease stage (Kiripolsky et al., 2017b; Robinson et al., 1998). Animals were euthanized using CO_2_ as the primary method followed by exsanguination as the secondary. The CO_2_ flow rate used was 14.2 L/min.

### Sex as a biologic variable

Both male and female mice were used to examine sexual dimorphism in pSD.

### Study approval

Animals were maintained in the Laboratory Animal Facility at the University of Buffalo and were housed and used in accordance with the institutional animal care and use committee (IACUC) of the University at Buffalo and US NIH guidelines.

### Tissue collection for histology

Submandibular salivary glands (SMGs), lacrimal glands, kidneys and lungs were harvested from female NOD.B10, NOD.B10^*Tlr7+/−*^ *,* and NOD.B10^*Tlr7−/−*^ mice. Tissue was also harvested from male NOD.B10 and NOD.B10^*Tlr7-/y*^ animals. Tissue was fixed in 10% formalin and paraffin embedded. H&E-stained sections were scanned using Aperio software and analysis was carried out using ImageJ. The percent of tissue occupied by lymphocytes was calculated as follows: (area of tissue occupied by inflammation/the total area of tissue examined) X 100, as previously described (Kiripolsky et al., 2021).

### Saliva collection and quantification

Saliva was collected and quantified as previously described (Kiripolsky et al., 2017b). Mice were given intraperitoneal injection of pilocarpine HCl (0.3 mg/100 μL PBS) (Sigma-Aldrich) to stimulate saliva production. Saliva was collected for 10 min following injection. Saliva was centrifuged briefly for 2 min at 16,000 g and the volume produced was quantified by pipette.

### Sera collection

Sera were collected from male and female mice by cardiac puncture immediately following euthanasia. Sera were incubated at room temperature for 2 h, and centrifuged for 20 min at 0.7 g. Sera were stored at −20°C until use.

### RNA isolation and quantitative reverse transcription polymerase chain reaction (qRT-PCR)

Spleens were harvested, snap frozen on dry ice, and stored at −80°C until use. Total RNA was extracted by resuspending the respective spleens in Trizol reagent (Thermo Fisher Scientific) using BioMashers (TaKaRa). The RNA was phase separated by chloroform and further isolated using the Direct-zol RNA Miniprep kit (Zymo Research). Isolated RNA was reverse transcribed using the iScript cDNA Synthesis kit (Bio-Rad) according to the manufacturer’s instructions and qRT-PCR was performed on a CFX96 Touch Real-Time PCR Detection System (Bio-Rad) using iQ SYBR Green Supermix (Bio-Rad). All qRT-PCR assays were performed in triplicates in at least three independent experiments. Relative expression values of each target gene were normalized to hypoxanthine guanine phosphoribosyltransferase (*Hprt*) expression. The following primers were used for qRT-PCR:

*Tlr7* forward 5′- CTG ACC GCC ACA ATC ACG TCA TG -3′.

*Tlr7* reverse 5′- GCT TGT CTG TGC AGTCCA CG -3′.

*Hprt* forward 5′- CCT CAT GGA CTG ATT ATG GAC AG -3′.

*Hprt* reverse 5′- TCA GCA AAG AAC TTA TAG CCC C -3′.

### Flow cytometry

Spleens and cervical lymph nodes (cLNs) were harvested and single cell suspensions were generated by mechanical dispersion. SMGs were harvested and mechanical and enzymatic dispersion was performed as previously published (Kiripolsky et al., 2020). SMGs derived from females were pooled from 2–4 animals. SMGs from males were examined individually, due to the more limited availability of males in the colony. Cell populations were identified as indicated using the following antibodies: B220 (clone RA3-6B2, BD Biosciences), CD23 (clone B3B4, Biolegend), CD21/35 (clone 7G6, BD Biosciences), T-bet (clone 4B10, BD Biosciences), CD11c (clone N418, BioLegend), CD11b (clone M1/70, BD Biosciences), CD5 (clone 53–7.3, BD Biosciences), CD1d (Clone 1B1, BD Biosciences), Fas (clone Jo2, BD Biosciences), GL7 (clone GL7, BioLegend), CD138 (clone 281–2, BD Biosciences), CD4 (clone GK1.5, BD Biosciences), CD8α (clone 53–6.7, BD Biosciences), CD44 (clone IM7, BD Biosciences), CD62L (clone MEL-14, BD Biosciences), CD69 (clone H1.2F3, Biolegend), Tlr7 (clone A94B10, BD Biosciences), and Tlr9 (clone J15A7, BD Biosciences). Data were acquired using a Fortessa (BD Biosciences) and quantified using Flow Jo (BD Biosciences).

### Western blotting

Spleens were harvested and mechanically dissociated. RBCs were removed using ACK lysing buffer (Gibco), and cells (1.0 × 10^7^) were lysed in RIPA buffer. A BioRad protein assay (BioRad Laboratories) was used to quantify protein and samples were solubilized using Laemmli buffer. Proteins were separated by electrophoresis and transferred to nitrocellulose membranes. Membranes were blotted overnight at 4°C with antibodies directed against Tlr7 (Cell Signaling Technology, clone E4J3Z) and β-actin (Cell Signaling Technology, clone 13E5). Membranes were incubated with HRP-conjugated secondary antibodies at RT for 1 h and developed using ECL reagents (BioRad Laboratories).

### Cell culture

Spleens were harvested and RBC lysis performed as described above. Splenocytes (5.0 × 10^6^) were cultured in either complete RPMI media containing 2% FBS, media containing LPS (*S*. *typhimurium*, 25 μg/mL), (Sigma-Aldrich) or media containing Imq (0.625 μg/mL) (InvivoGen) for 24 h prior to assay. Supernatants were harvested and stored at −20°C until use. Cells were assayed for activation by flow cytometry, as described below.

### ELISAs

IgM, IgG (Invitrogen), and β2-microglobulin ELISAs (LifeSpan BioSciences) were carried out using serially diluted sera. IL-6 ELISAs (Invitrogen) were performed on cell culture supernatants. ELISAs were performed in accordance with manufacturer instructions and all samples were analyzed in duplicate.

### HEp-2 staining and autoantigen arrays

HEp-2 assays were carried out as previously described (Kiripolsky et al., 2021). Since females had more total serum IgG than males, all female sera were assessed at a dilution of 1:50, while male sera were used at a dilution of 1:20. Autoantigen array analyses were performed by the UT Southwestern Genomics and Microarray Core facility as previously described (Punnanitinont et al., 2024).

### Statistics

The comparisons of interest regarding 2 groups are: NOD.B10 males vs. NOD.B10 females and NOD.B10 males vs. NOD.B10^*Tlr7-/y*^ males. For the females, the comparison of interest was between the 3 groups: NOD.B10, NOD.B10^*Tlr7+/−*^ and NOD.B10^*Tlr7−/−*^. A Mann-Whitney test at level 0.05 was used to compare groups of 2 and ANOVA with *post hoc* Tukey test at level 0.05 was applied to assess the female animals (NOD.B10, NOD.B10^*Tlr7+/−*^ and NOD.B10^*Tlr7−/−*^) (GraphPad Software, version 10.0.2). Autoantigen array data were analyzed using previously described methods (Punnanitinont et al., 2022). We analyzed all of the array data to identify autoantibodies that differentially enriched between males and female NOD.B10 mice. We focused on the 27 ANA-specific autoantigens to detect differences between *Tlr7*-decificient animals and sex-matched *Tlr7*-sufficient controls. For each ANA-specific autoantigen in IgM, we performed a 2-sample 2-sided *t*-test to assess enrichment in male NOD.B10 mice as compared to NOD.B10^*Tlr7-/y*^ males. We used the p. adjust R function in the R Stats package (R Foundation for Statistical Computing, version 4.3.1) to adjust the P values in order to control the false discovery rate (FDR) across the ANA-specific autoantigens. The method proposed by Benjamini and Hochberg was used to control the false discovery rate (Benjamini and Yekutieli, 2001). An autoantigen was deemed significant if the corresponding adjusted P-value was <0.10.

### Data availability

The autoantigen array data are deposited in the Gene Expression Omnibus database under the following accession number: GSE255215.

## Results

### Tlr7 is ablated in NOD.B10 mice

**FIGURE 1:**
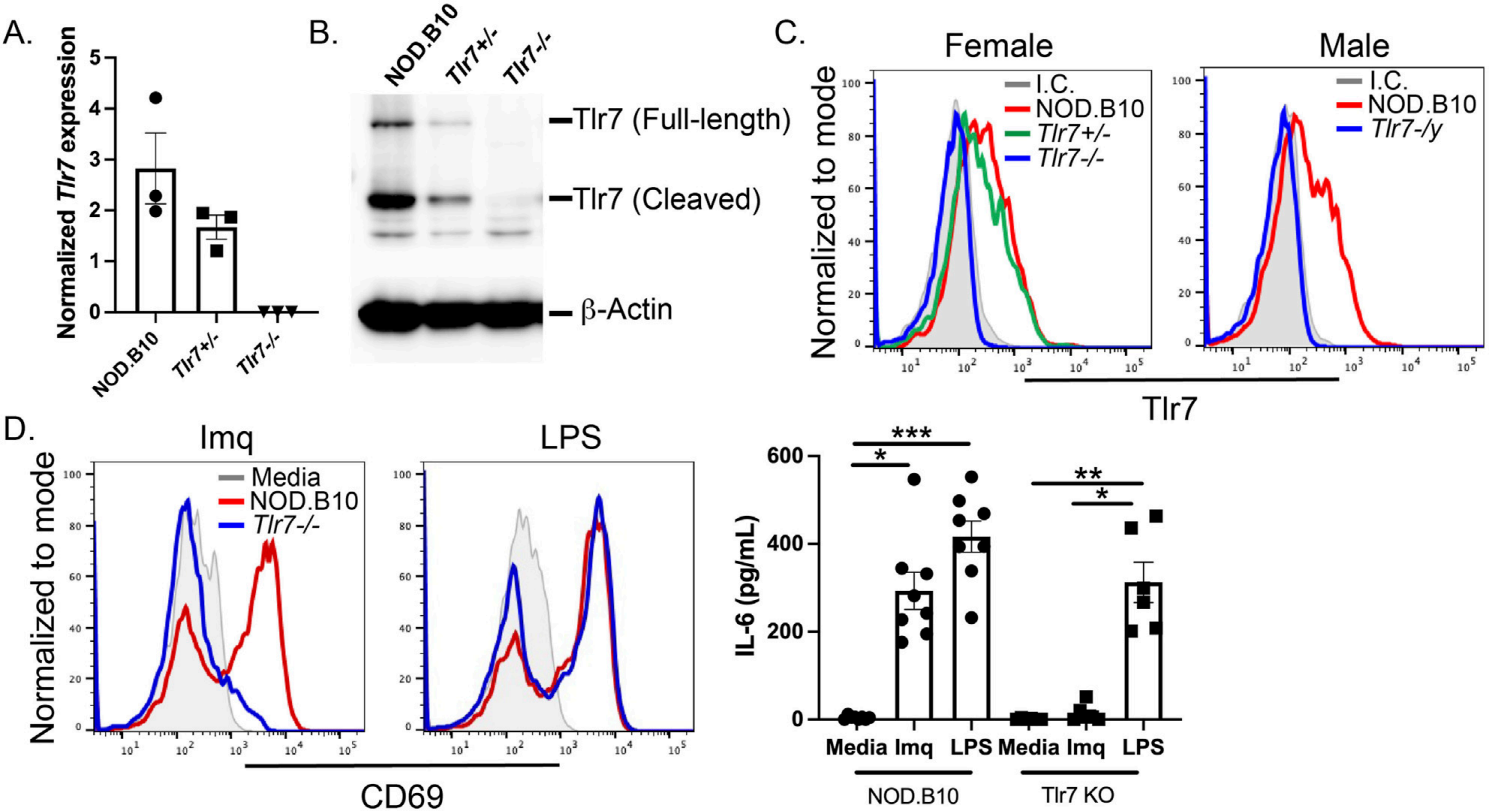
Tlr7 is ablated in male and female NOD.B10 mice. Spleens were harvested from male and female mice and **(A)** qRT-PCR was performed on female NOD.B10, NOD.B10^*Tlr7+/−*^ *,* and NOD.B10^*Tlr7−/−*^ spleens (n = 3 each). **(B)** Western blotting was performed on male and female mice. Representative data from one female NOD.B10, NOD.B10^*Tlr7+/−*^ *,* and NOD.B10^*Tlr7−/−*^ spleen is shown. **(C)** Flow cytometry was performed to assess expression of Tlr7 in B cells from NOD.B10 (n = 11), NOD.B10^*Tlr7+/−*^ (n = 9), and NOD.B10^*Tlr7−/−*^ females (n = 13). NOD.B10 (n = 9) and NOD.B10^*Tlr7-/y*^ males (n = 9) were also assessed. Representative histogram plots from one animal of each sex and strain is shown. **(D)** Splenocytes were harvested from NOD.B10 females (n = 6) and NOD.B10 males (n = 2). Spleens from NOD.B10^*Tlr7−/−*^ females (n = 3) and NOD.B10^*Tlr7-/y*^ (n = 3) mice were also harvested and cultured in media alone, in Imq, or in LPS. Supernatants were harvested and IL-6 was quantified by ELISA. Cells were harvested and CD69 expression was assessed by flow cytometry. Representative histogram plots from one animal of each strain are shown from each treatment condition. Horizontal lines represent mean and standard error of the mean (SEM), (*p < 0.05, **p < 0.01, ***p < 0.0001 for the hypothesis tests described in the statistical section).

To ablate Tlr7 expression systemically in NOD.B10 mice, NOD.B10 animals were bred to B6.129S1-*Tlr7* ^*tm1Flv*^/J mice for 7 generations, as described in the Methods section. Spleens were harvested from female NOD.B10, NOD.B10^*Tlr7+/−*^ *,* and NOD.B10^*Tlr7−/−*^ mice and NOD.B10 and NOD.B10^*Tlr7-/y*^ males. Quantitative reverse-transcription polymerase chain reaction and western blotting performed on splenic tissue demonstrated ablation of Tlr7 mRNA and protein expression in the NOD.B10^*Tlr7−/−*^ mice (Figures 1A, B, respectively). This observation was consistent for both full-length and cleaved forms of the protein (Figure 1B). Flow cytometry was also performed to confirm ablation of Tlr7 in splenic B cells. NOD.B10 female *Tlr7* heterozygous mice had a decreased percentage of Tlr7+ B cells as compared to sex-matched NOD.B10 females. As expected, B cell Tlr7 expression was ablated in the NOD.B10^*Tlr7−/−*^ females (Figure 1C). Similar results were observed in male animals, as NOD.B10^*Tlr7-/y*^ males showed negligible expression of Tlr7 in the B cell compartment (Figure 1C).

Corroborative functional studies confirmed the absence of Tlr7 expression. Spleens were harvested from male and female NOD.B10 mice and their *Tlr7*-deficient counterparts. Splenocytes were cultured in media alone, or in media containing the Tlr7 agonist Imiquimod (Imq) or the Tlr4 agonist lipopolysaccharide (LPS). Cells were assayed by flow cytometry and CD69 was used to assess B cell activation. As expected, both Tlr7-sufficient and deficient B cells upregulated CD69 in response to LPS (Figure 1D). B cells derived from mice lacking Tlr7 expression were unresponsive to Imq, while splenocytes from NOD.B10 mice showed robust upregulation of CD69 in response to Imq stimulation (Figure 1D). Finally, supernatants from the cultured cells were harvested and IL-6 secretion was quantified by ELISA. IL-6 was secreted by splenocytes from all strains stimulated with LPS, while supernatants derived from Tlr7-deficient splenocytes secreted negligible amounts of IL-6. Finally, splenocytes from NOD.B10 females and males secreted high levels of IL-6 following Imq stimulation (Figure 1D). Altogether, these data confirm ablation of Tlr7 in female and male pSD mice.

### NOD.B10 mice display sex-biased lacrimal gland inflammation and Tlr7 ablation enhances salivary inflammation in males and females

**FIGURE 2:**
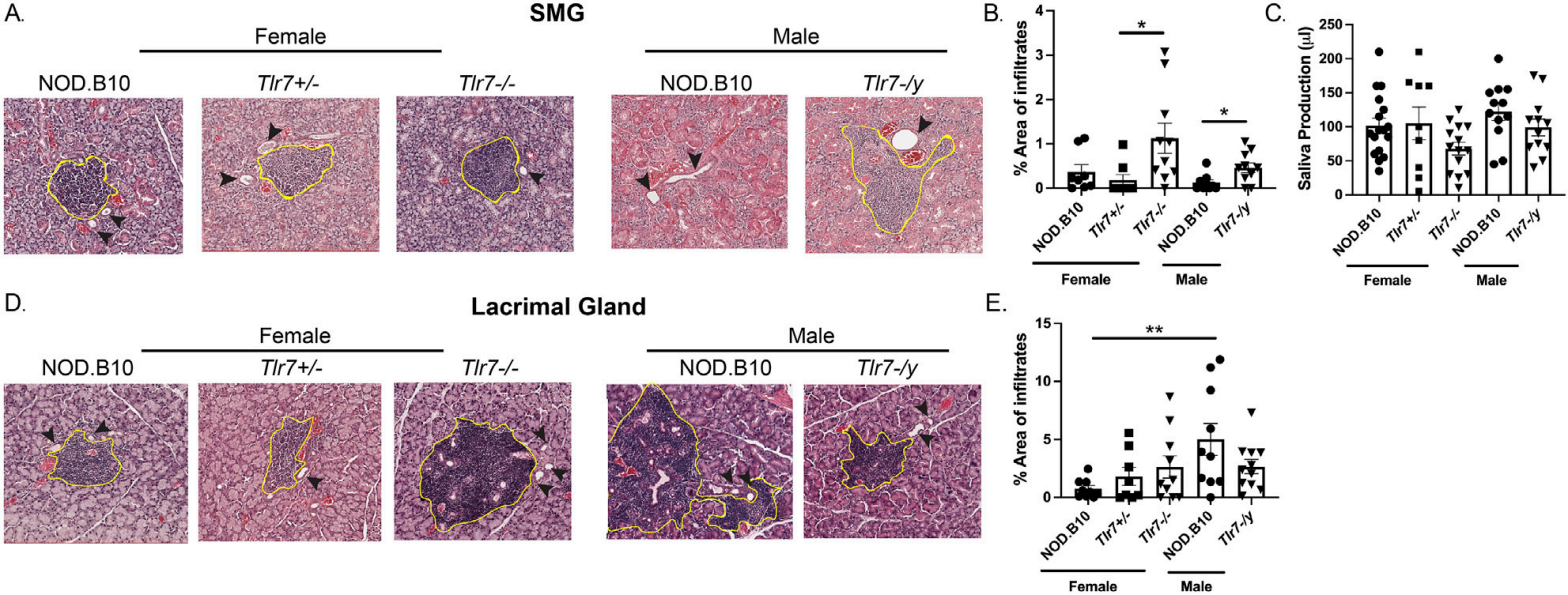
Lacrimal inflammation is heightened in male NOD.B10 mice and Tlr7 protects against inflammation in female and male SMG tissue. SMG and lacrimal tissues were harvested from NOD.B10 (n = 9), NOD.B10^*Tlr7+/−*^ (n = 8), and NOD.B10^*Tlr7−/−*^ (n = 10) females. Glands were also harvested from NOD.B10 (n = 10) and NOD.B10^*Tlr7-/y*^ (n = 10) males. **(A)** Representative H&E-stained SMG images are shown. The area of tissue occupied by lymphocytes in the SMG is shown for **(B)** NOD.B10, NOD.B10^*Tlr7+/−*^ *,* and NOD.B10^*Tlr7−/−*^ females and NOD.B10 and NOD.B10^*Tlr7-/y*^ males. **(C)** Saliva production for NOD.B10 (n = 17), NOD.B10^*Tlr7+/−*^ (n = 9), and NOD.B10^*Tlr7−/−*^ females (n = 14) and NOD.B10 (n = 12) and NOD.B10^*Tlr7-/y*^ males (n = 12) is shown. **(D)** Representative H&E-stained lacrimal gland images are shown. The area of tissue occupied by lymphocytes in lacrimal tissue is shown for **(E)** NOD.B10, NOD.B10^*Tlr7+/−*^ *,* and NOD.B10^*Tlr7−/−*^ females and NOD.B10 and NOD.B10^*Tlr7-/y*^ males. Horizontal lines represent mean and SEM, (*p < 0.05, **p < 0.01 for the hypothesis tests described in the statistical section).

Exocrine tissue was harvested from male and female mice of each strain and the percentage of tissue occupied by lymphocytes was quantified (Figure 2). Analysis of SMG tissue revealed no differences in the amount of inflammation between males and females (Figure 2B). SMG tissue from female NOD.B10, NOD.B10^*Tlr7+/−*^ and NOD.B10^*Tlr7−/−*^ mice was also examined, and SMG lymphocytic infiltration was increased in NOD.B10^*Tlr7−/−*^ females and compared to NOD.B10^*Tlr7+/−*^ controls (*p* = 0.01) (Figure 2B). SMG tissue from male NOD.B10 and NOD.B10^*Tlr7-/y*^ animals was also assessed, and *Tlr7*-deficient males exhibited enhanced salivary inflammation as compared to the male NOD.B10 parental strain (*p* = 0.03) (Figure 2B). To assess the functional consequences of Tlr7 ablation in salivary tissue, stimulated saliva was collected from all strains at the clinical disease stage (26 weeks of age). We found no differences in saliva production between NOD.B10 males and females or between the female and male *Tlr7*-deficient strains (Figure 2C). Of note, both male and female *Tlr7*-deficient mice had similar weights when compared to sex-matched parental controls (data not shown).

Lacrimal tissue was also examined, and robust lymphocytic infiltration was observed in male NOD.B10 mice as compared to females (*p* = 0.006) (Figures 2D, E), and this was consistent with a prior study (Mauduit et al., 2022). We then examined lacrimal inflammation in both female and male Tlr7-deficient strains, and found no differences when *Tlr7* was ablated in either females or males (Figure 2E). Therefore, while we observed no sex-biased inflammation in SMG tissue, lacrimal inflammation was increased in male NOD.B10 mice as compared to females. Moreover, both males and females show heightened salivary inflammation in the absence of *Tlr7*, while *Tlr7*-deficient mice showed no differences in dacryoadenitis as compared to *Tlr7*-sufficient controls.

### Immune populations are altered in SMG tissue in Tlr7-deficient males and females

**FIGURE 3:**
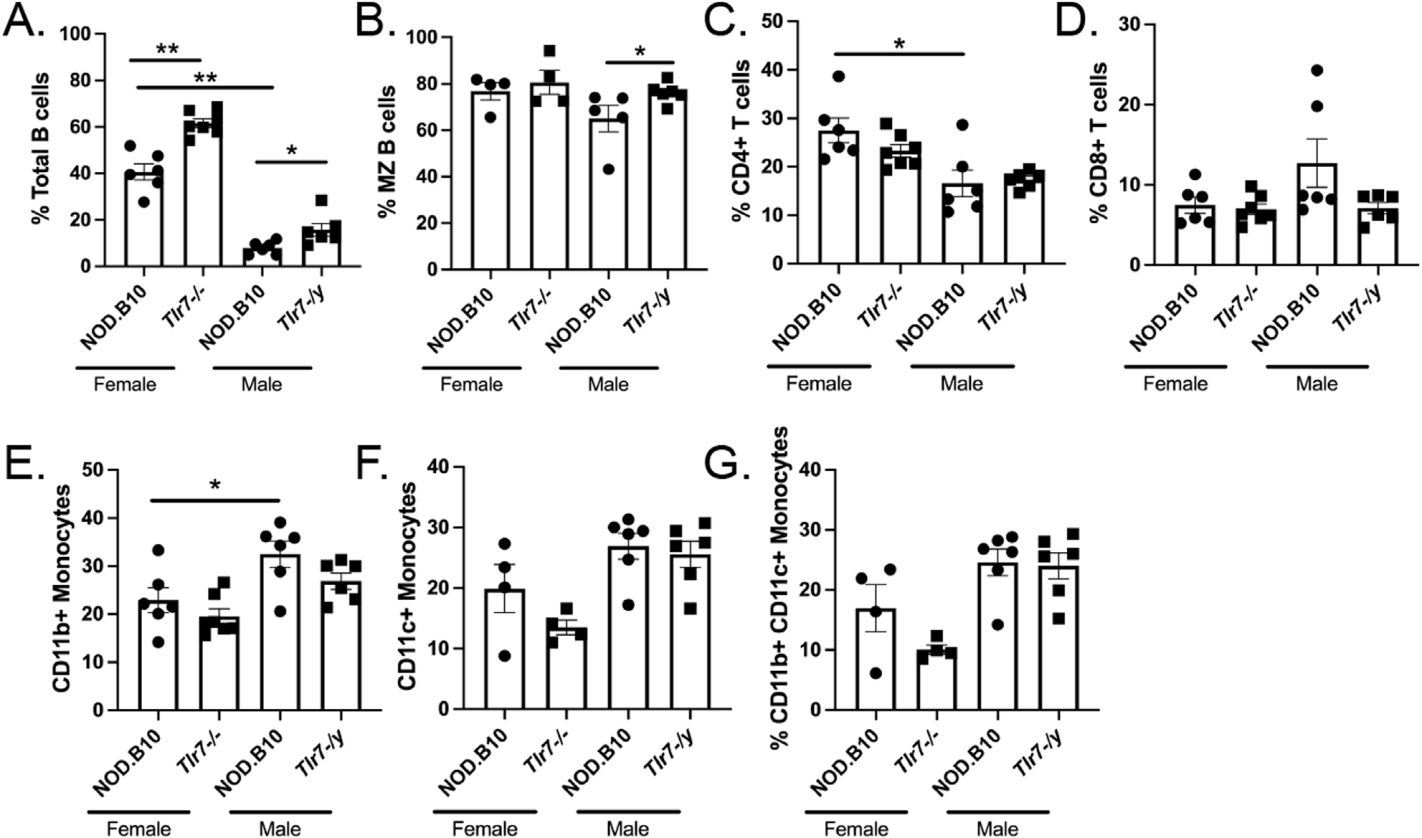
Immune populations are altered in the salivary tissue of male and female *Tlr7*-deficient mice. SMGs were harvested from NOD.B10 females and NOD.B10^*Tlr7−/−*^ age- and sex-matched mice (n = at least 4 pooled samples each). SMGs were also harvested from NOD.B10 males (n = 6) and NOD.B10^*Tlr7-/y*^ animals (n = 6) and flow cytometry was performed to identify **(A)** Total B cells (B220+), **(B)** MZ B cells (B220+ CD23^−^ CD21^+^) **(C)** CD4^+^ T cells, **(D)** CD8^+^ T cells, **(E)** CD11b+ monocytes (CD11b+ B220-), **(F)** CD11c+ monocytes (CD11c+ B220-), and **(G)** CD11b+ CD11c+ monocytes (CD11c+ CD11b+ B220-). Horizontal lines represent mean and SEM, (*p < 0.05, **p < 0.01 for the hypothesis tests described in the statistical section).

Since sialadenitis was increased in both *Tlr7*-deficient males and females, we sought to analyze the infiltrates in SMG tissue in greater depth. We harvested SMGs from female and male NOD.B10 mice and *Tlr7*-deficient age-and sex-matched animals and performed flow cytometry to assess both innate and adaptive immune populations in salivary tissue (Figure 3). SMG flow cytometry gating strategies are shown in Supplementary Figure 1. We first compared SMGs from female and male NOD.B10 mice. We found that NOD.B10 females had expanded total B cell and CD4^+^ T cell populations as compared to males (*p* = 0.002 and *p* = 0.03, respectively) (Figures 3A, C), while NOD.B10 males had a greater percentage of CD11b+ monocytes as compared to females (*p* = 0.04) (Figure 3E). There were no differences in the percentages of marginal zone (MZ) B cells, CD8^+^ T cells, and CD11c+ and CD11b+ CD11c+ monocytes between males and females (Figures 3B, D, F, G, respectively).

We next compared immune populations in SMGs derived from female NOD.B10 mice to those from their *Tlr7*-deficient counterparts, and we found that the percentage of total B cells was increased in NOD.B10^*Tlr7−/−*^ females (*p* = 0.001) (Figure 3A). Ablation of *Tlr7* did not alter the percentages of MZ B cells, T cells, or monocytes in SMG tissue in females (Figures 3B–G). We next examined immune subsets in SMG tissue derived from NOD.B10^*Tlr7-/y*^ mice and compared these to those from NOD.B10 males. Our data revealed that the percentage of total and MZ B cells increased in *Tlr7*-deficient males as compared to their NOD.B10 counterparts (*p* = 0.02 and *p* = 0.02, respectively) (Figures 3A, B). We did not observe any differences on the percentages of T cells or monocytes in SMG tissue between the NOD.B10 and NOD.B10^*Tlr7-/y*^ males (Figures 3C–G).

Of note, we also performed experiments to examine ABCs in the SMG tissue of each of the groups. We were able to identify this population (data not shown), although we did not detect enough cells to perform meaningful analyses of this subset. While ABCs exist in SMG tissue of NOD.B10 mice and their *Tlr7*-deficient counterparts, further studies with greater numbers of pooled SMGs from each strain are needed to quantify this population. Thus, innate and adaptive immune subsets differ between males and females in SMG tissue, and B cell subsets are altered in *Tlr7*-deficient males and females as compared to age- and sex-matched NOD.B10 animals.

### Females display worse nephritis as compared to males, and Tlr7 protects against pulmonary inflammation in males

**FIGURE 4:**
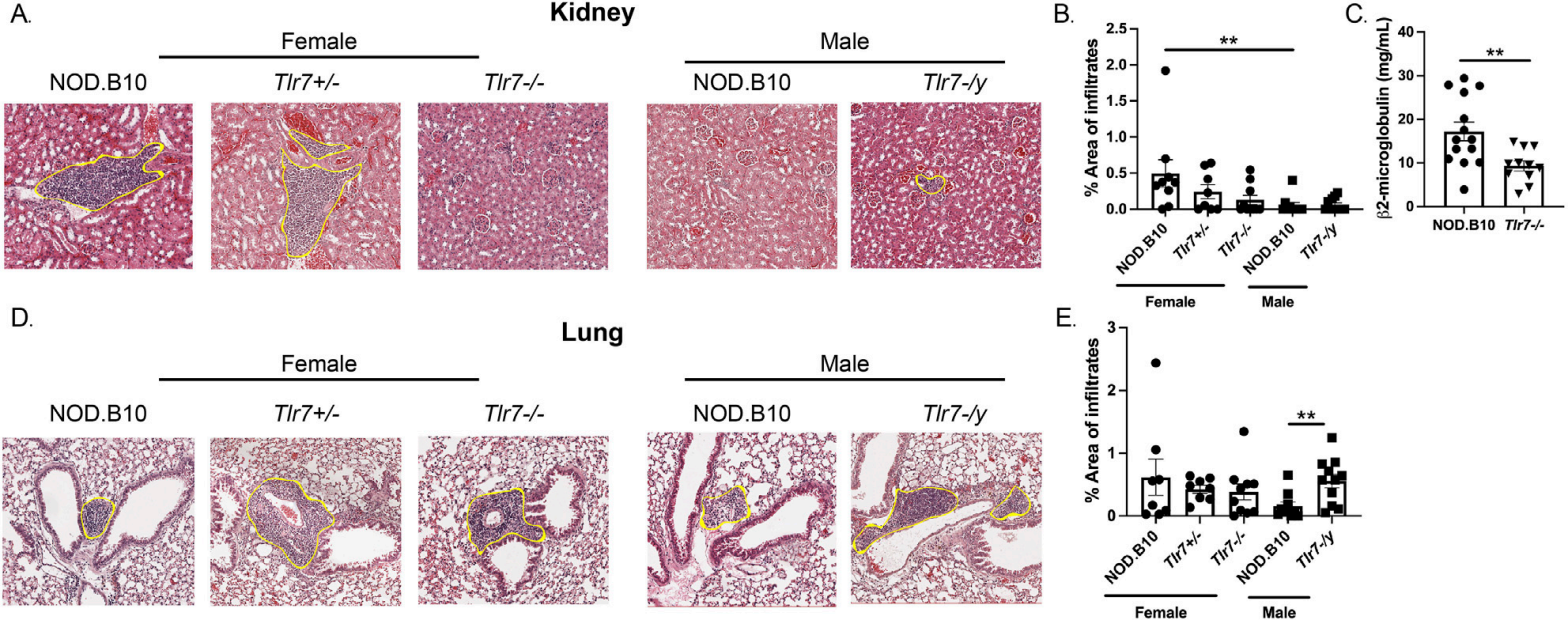
Nephritis is diminished in male pSD mice and ablation of Tlr7 exacerbates lung inflammation in males. Kidneys and lungs were harvested from NOD.B10 (n = 9), NOD.B10^*Tlr7+/−*^ (n = 8), and NOD.B10^*Tlr7−/−*^ (n = 10) females. Tissues were also harvested from NOD.B10 (n = 10) and NOD.B10^*Tlr7-/y*^ (n = 11) males. **(A)** Representative H&E-stained images are shown. The area of tissue occupied by lymphocytes in kidney tissue is shown for **(B)** NOD.B10, NOD.B10^*Tlr7+/−*^ *,* and NOD.B10^*Tlr7−/−*^ females and NOD.B10 and NOD.B10^*Tlr7-/y*^ males. **(C)** Sera were harvested from NOD.B10 (n = 14) and NOD.B10^*Tlr7−/−*^ females (n = 11). Levels of β2-microglobulin were quantified by ELISA. **(D)** Representative H&E-stained lung tissue images are shown. The area of tissue occupied by lymphocytes in lung tissue is shown for **(E)** NOD.B10, NOD.B10^*Tlr7+/−*^ *,* and NOD.B10^*Tlr7−/−*^ females and NOD.B10 and NOD.B10^*Tlr7-/y*^ males. Horizontal lines represent mean and SEM, (**p < 0.01 for the hypothesis tests described in the statistical section).

We next focused our studies on systemic disease manifestations and examined inflammation in both kidney and lung tissue (Figure 4). We found that NOD.B10 females displayed greater inflammation in kidney tissue as compared to males (*p* = 0.006) (Figures 4A, B). Additionally, ablation of *Tlr7* diminished inflammation in females as compared to the NOD.B10 parental strain, although this did not reach statistical significance using the tests described in the Methods section. There was a significant decrease in nephritis, however, in the NOD.B10^*Tlr7−/−*^ females and compared to sex-matched NOD.B10 controls when we compared the two groups using the Mann Whitney test (*p* = 0.04) (Figure 4B). Ablation of Tlr7 did not alter the degree of nephritis male mice (Figure 3B). We then assessed β2-microglobulin as an indicator of kidney function in NOD.B10 and NOD.B10^*Tlr7−/−*^ females. Our data revealed that NOD.B10 females had higher levels of β2-microglobulin in sera as compared to their *Tlr7*-deficient counterparts (*p* = 0.003) (Figure 4C).

Next, lung inflammation was quantified in NOD.B10 females and males, and levels were similar between the sexes (Figures 4D, E). We then examined NOD.B10^*Tlr7−/−*^ females, and found no differences in pulmonary inflammation as compared to that of NOD.B10 or NOD.B10^*Tlr7+/−*^ females (Figures 4D, E). Finally, we compared pneumonitis between NOD.B10 and NOD.B10^*Tlr7-/y*^ males. Our data revealed that *Tlr7*-deficient male mice had heightened tissue inflammation as compared to the NOD.B10 parental strain (*p* = 0.008) (Figures 4D, E). Thus, sex-biased inflammation is observed in kidney tissue in females in the context of pSD. Moreover, Tlr7 exacerbates nephritis in females, while Tlr7-mediated signals protect against pulmonary inflammation in pSD males.

### Females pSD mice have expanded B and T populations associated with disease in secondary lymphoid tissue

**FIGURE 5:**
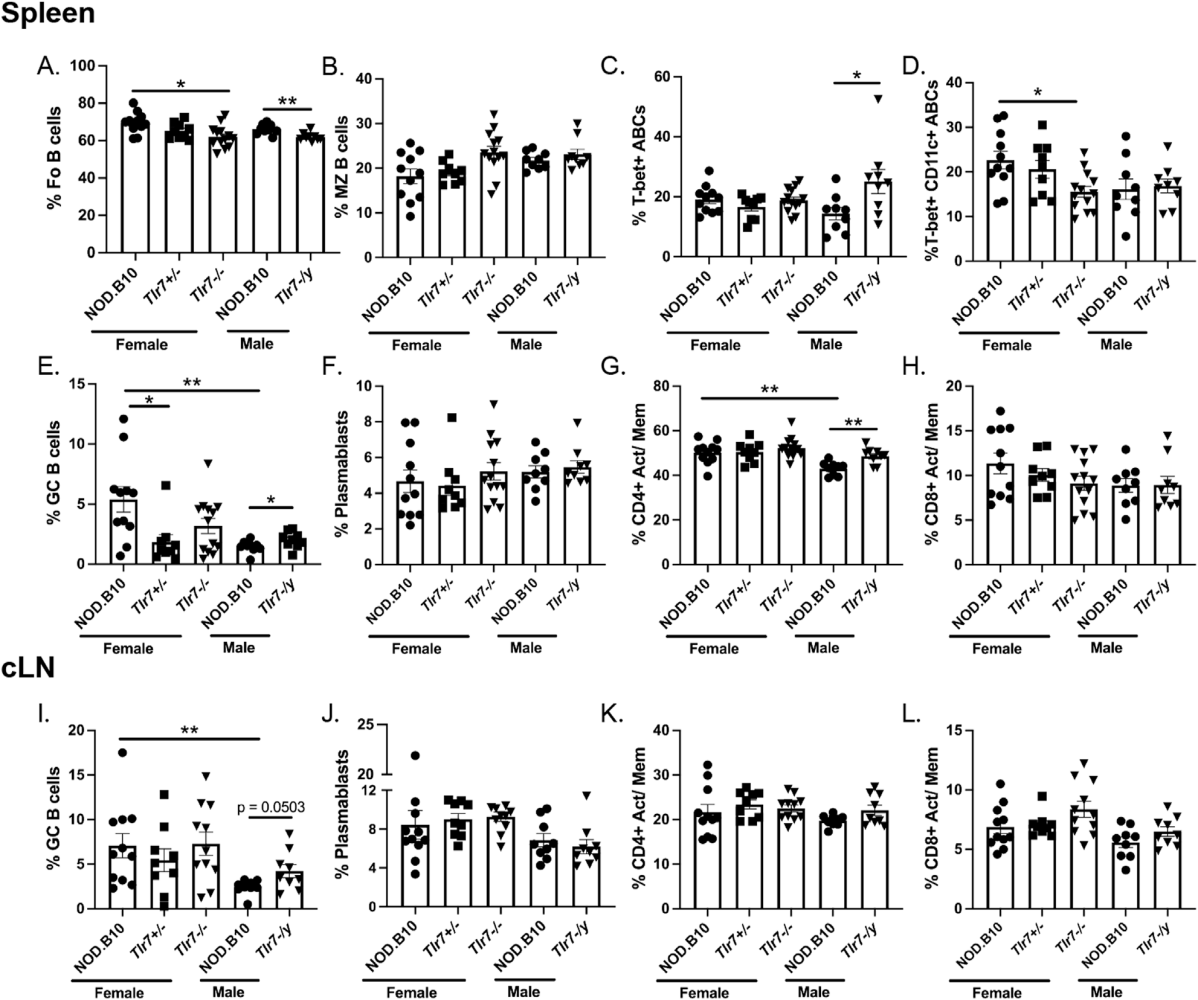
NOD.B10 females exhibit heightened immune activation in secondary lymphoid tissues as compared to males and ablation of Tlr7 in males and females has dichotomous effects. Spleens and cLNs were harvested from NOD.B10 female (n = 11) and male mice (n = 8) and from NOD.B10^*Tlr7+/−*^ (n = 9), NOD.B10^*Tlr7−/−*^ (n = 13), and NOD.B10^*Tlr7-/y*^ mice (n = 9). Flow cytometry was performed to quantify the following immune populations in the spleen: **(A)** Fo B cells (B220+ CD23^+^CD21^lo/-^), **(B)** MZ B cells (B220+ CD23^−^ CD21^+^), **(C)** T-bet + ABCs (B220+ CD23^−^ CD21^−^ T-bet+ CD11c+), **(D)** T-bet+ CD11c+ ABCs (B220+ CD23^−^ CD21^−^ T-bet+ CD11c+) **(E)** GC B cells (B220+ Fas + GL7+), **(F)** Plasmablasts (B220+ CD138+), **(G)** Activated/memory (Act/Mem) CD4^+^ T cells (CD4^+^ CD44^+^ CD62L-) and **(H)** Activated/memory CD8^+^ T cells (CD8^+^ CD44^+^ CD62L-). Similar populations were quantified in cLNs as follows: **(I)** GC B cells, **(J)** Plasmablasts **(K)** Activated/memory CD4^+^ T cells, and **(L)** Activated/memory CD8^+^ T cells. Horizontal lines represent mean and SEM, (*p < 0.05, **p < 0.01 for the hypothesis tests described in the statistical section).

We next sought to determine whether NOD.B10 females displayed expansion of distinct immune populations implicated in pSD pathogenesis as compared to males. Towards this end, we harvested spleens and cLNs from NOD.B10 mice at the clinical disease stage and assessed B and T cell populations (Figure 5). Flow cytometry gating strategies are shown in Supplementary Figure 2. We first examined splenic populations and found no differences in total B cells or CD4^+^ or CD8+T cell populations (data not shown). We also examined B cell subsets and found no differences in either the percentage of follicular (FO) or MZ B cells between males and females (Figures 5A, B, respectively). T-bet + ABCs and plasmablasts were also similar between the sexes (Figures 5C, F, respectively). T-bet+ CD11c+ ABCs tended to be diminished in male NOD.B10 mice, although this difference did not reach significance (*p* = 0.0503) (Figure 5D). We found, however, that GC B cells were diminished in NOD.B10 males as compared to females (*p* = 0.006) (Figure 5E). Finally, we assessed activated/memory T cells populations in the spleen. Activated/memory CD4^+^ T cells were expanded in females (*p* = 0.001), although the activated/memory CD8^+^ T cell populations were unchanged between the sexes (Figures 5G, H, respectively).

We next quantified cLN immune populations. Similar to the spleen, our data revealed no differences in total B cell or CD4^+^ or CD8^+^ T cell populations (data not shown). Additionally, the percentage of plasmablasts and activated/memory CD4^+^ and CD8^+^ T cells were similar between males and females (Figures 5J–L, respectively). GC B cells, however, were increased in the cLNs derived from female NOD.B10 mice as compared to males (*p* = 0.003) (Figure 5I). Altogether, these studies identify expansion of GC B cells and CD4^+^ activated/memory T cells in secondary lymphoid tissue of pSD females as compared to males.

### Ablation of Tlr7 reduces splenic immune populations associated with pSD in females, while male pSD mice that lack Tlr7 show expansion of B and T subsets implicated in disease

We next sought to determine if immune populations were altered in Tlr7-deficient female and male mice. We first examined spleens of female NOD.B10, NOD.B10^*Tlr7+/−*^ and NOD.B10^*Tlr7−/−*^ animals. While we found no differences in the percentage of total B cells, MZ B cells, T-bet + ABCs, or plasmablasts (data not shown and Figures 5B, C, F), both FO and T-bet+ CD11c+ ABCs were diminished in NOD.B10^*Tlr7−/−*^ females as compared to NOD.B10 controls (*p* = 0.01 and *p* = 0.03, respectively) (Figures 5A, D). The percentage of GC B cells was decreased in NOD.B10^*Tlr7+/−*^ mice as compared to the NOD.B10 parental strain (*p* = 0.04), although no difference in this population was observed between NOD.B10 and NOD.B10^*Tlr7−/−*^ animals (Figure 5E). We also examined total and activated/memory CD4^+^ and CD8^+^ T cells populations and found no differences among the female strains in the T cell compartment (data not shown and Figures 5G, H, respectively). Finally, we found no differences among any of the female cLN immune populations examined (Figures 5I–L).

We then carried out analogous studies in splenic tissue derived from male NOD.B10 and NOD.B10^*Tlr7-/y*^ mice. We found that the percentage of total B cells was similar between the 2 strains (data not shown), although FO B cells were decreased in *Tlr7*-deficient males as compared to NOD.B10 mice (*p* = 0.006) (Figure 5A). In contrast, our data revealed that distinct immune cell populations associated with disease were expanded in *Tlr7*-deficient males, namely, T-bet + ABCs, GC B cells, and CD4^+^ activated/memory T cells (*p* = 0.02, *p* = 0.04, and *p* = 0.008, respectively) (Figures 5C, E, G). Of note, we observed no differences in splenic T-bet+ CD11c+ ABCs, plasmablasts, or CD8^+^ activated/memory T cells between NOD.B10 and NOD.B10^*Tlr7-/y*^ males (Figures 5D, F, H, respectively). Lastly, we examined cLN immune population in male strains. Similar to females, we observed no differences (Figures 5I–L). Although GC B cells tended to increase in NOD.B10^*Tlr7-/y*^ males as compared to the NOD.B10 parental strain, this difference was not significant (*p* = 0.0503) (Figure 5I). Of note, we also analyzed B regulatory subsets (B220+ CD1d+ CD5^+^) in spleens derived from female NOD.B10 (n = 11), NOD.B10^*Tlr7+/−*^ (n = 9) and NOD.B10^*Tlr7−/−*^ females (n = 13). We also examined this population in *Tlr7*-sufficient and deficient pSD males (n = 9 and 10, respectively). We found that this subset was present in relatively low abundance (1%–3% of total splenic B cells) in all of the mice examined, and there were no significant differences observed among the sexes or strains (data not shown). Altogether, these results demonstrate that Tlr7 ablation tends to diminish immune populations associated with pSD in the spleens of females, while pSD males that lack Tlr7 show expansion of splenic B and T subsets that mediate disease.

### Total serum IgG is increased in NOD.B10 females as compared to males and IgG titers are decreased in Tlr7-deficient females as compared to Tlr7-sufficient controls

**FIGURE 6:**
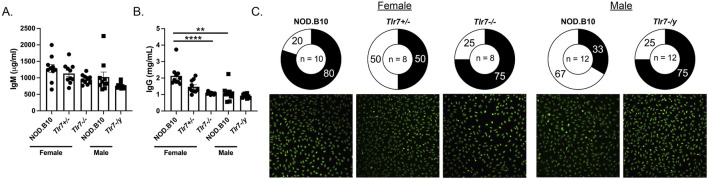
Female NOD.B10 mice have increased serum IgG titers as compared to males and ablation of Tlr7 diminishes IgG in females, although ANA-specific IgG was similar between Tlr7-deficient mice and sex-matched controls. Sera from NOD.B10 males and females, and NOD.B10^*Tlr7+/−*^, NOD.B10^*Tlr7−/−*^, and NOD.B10^*Tlr7-/y*^ mice were harvested (n = 10 each) and ELISAs for **(A)** IgM and **(B)** IgG were performed. Horizontal lines represent mean and SEM, (**p < 0.01, ****p < 0.0001 for the hypothesis tests described in the statistical section). **(C)** HEp-2 staining was performed on sera from NOD.B10 (n = 10), NOD.B10^*Tlr7+/−*^ (n = 8), NOD.B10^*Tlr7−/−*^ (n = 8) females and NOD.B10 (n = 12) and NOD.B10^*Tlr7-/y*^ males (n = 12). The pie charts show percentages of sera showing negligible fluorescence (clear regions). The shaded regions represent sera with HEp-2 reactive IgG autoantibodies. Images from one animal from each group are shown.

We next evaluated total IgM and IgG titers in the male and female animals. While there was no difference in IgM between males and females (Figure 6A), IgG titers were elevated in sera derived from female mice as compared to males (*p* = 0.0005) (Figure 6B). We next assessed IgM and IgG levels in NOD.B10, NOD.B10^*Tlr7+/−*^ and NOD.B10^*Tlr7−/−*^ females. IgM levels were similar between each of the strains (Figure 6A), while IgG was decreased in the *Tlr7*-deficient mice as compared to the NOD.B10 counterparts (*p* < 0.0001) (Figure 6B). In contrast, serum IgM and IgG levels were similar between NOD.B10 and NOD.B10^*Tlr7-/y*^ males (Figures 6A, B).

### Select autoantigens are enriched in a sex-specific manner in pSD mice and NOD.B10^*Tlr7-/y*^ mice have diminished ANA-specific IgM as compare to Tlr7-sufficient controls

**FIGURE 7:**
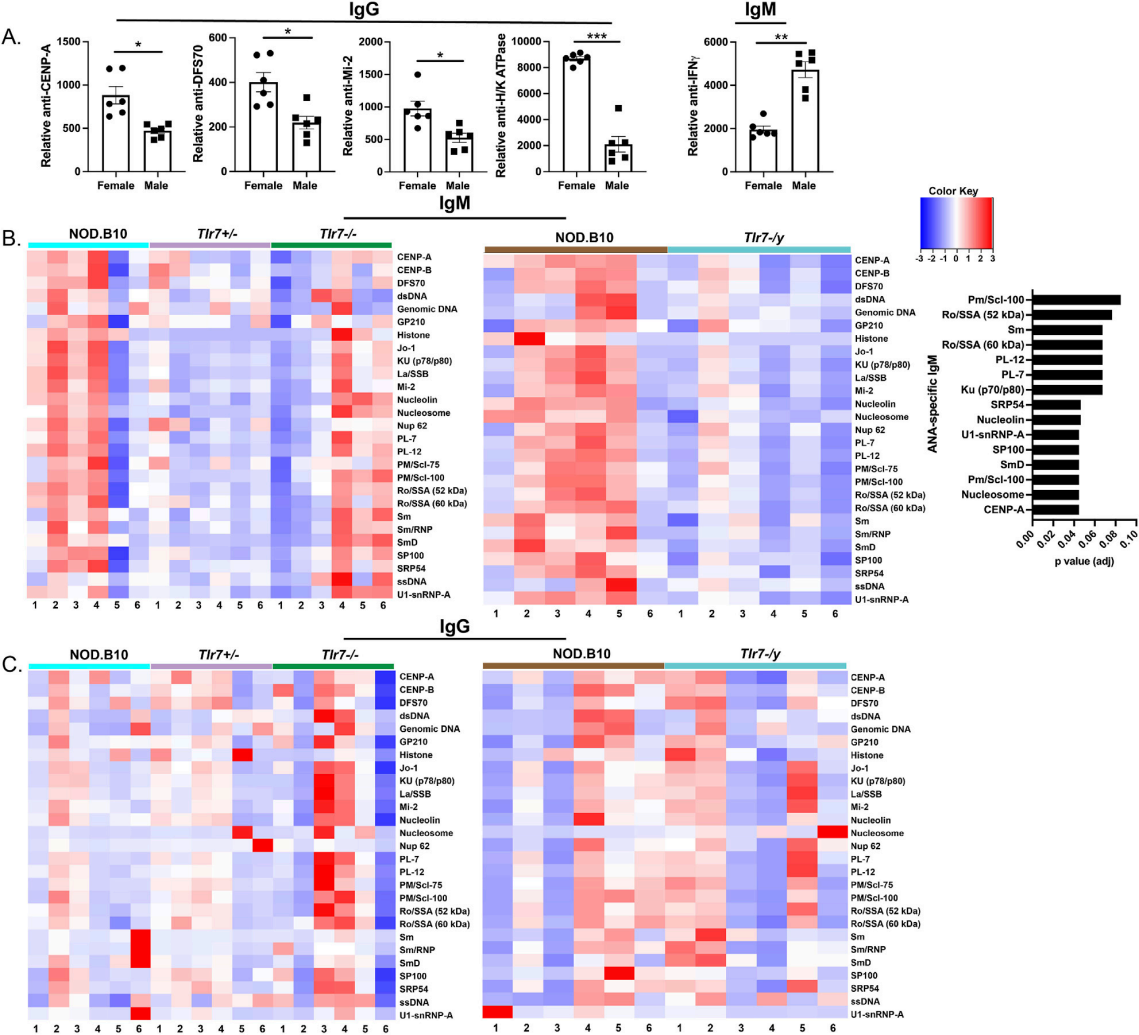
Sera from NOD.B10 males shows enriched reactivity for ANA-specific IgM autoantigens as compared to sex-matched *Tlr7*-deficient mice. Sera from NOD.B10 males and females, and NOD.B10^*Tlr7+/*^, NOD.B10^*Tlr7−/−*^, and NOD.B10^*Tlr7-/y*^ mice were harvested (n = 6 each) and autoantigen arrays were performed. **(A)** Normalized net signal intensity values for IgM and IgG autoantibodies differentially expressed in a sex-biased manner are shown. Horizontal lines represent mean and SEM (*adj p-value < 0.1, **adj p-value < 0.05, ***adj p-value < 0.01 for the hypothesis tests described in the statistical section). **(B)** Heatmaps showing ANA-specific IgM are shown for female (left panel) and male mice (middle panel). Autoantibodies that are enriched in male NOD.B10 mice as compared to NOD.B10^*Tlr7-/y*^ mice are shown (right panel). **(C)** Heatmaps illustrating ANA-specific IgG reactivity are shown for female (left panel) and male mice (right panel).

We performed HEp-2 staining to assess autoreactivity in the IgG compartment in each of the groups. We observed no difference in the percentage of animals that had detectable IgG-specific ANA in females, as 80% of NOD.B10 females (8/10), 50% of NOD.B10^*Tlr7+/−*^ females (4/8) and 75% of NOD.B10^*Tlr7−/−*^ females (6/8) had detectable ANAs (Figure 6C). Our data in males, however, suggested that ANAs may be enriched in *Tlr7*-deficient animals, as 33% of NOD.B10 males displayed ANAs (4/12), while 75% of NOD.B10^*Tlr7-/y*^ mice detectable ANAs (9/12) (Figure 6C). Of note, we were unable to compare our HEp-2 findings between males and females, because we used a lower concentration of sera in the female studies as compared to the males as described in the Methods. To extend these findings, we then performed autoantigen arrays to assess autoreactivity within the sera among groups. We first compared sera from male and female NOD.B10 mice. Our data revealed that select ANA-specific IgG autoantibodies directed against CENP-A (*p* = 0.09), DSF-70 (*p* = 0.09) and Mi-2 (*p* = 0.09) were enriched in females as compared to males (Figure 6A). Additionally, anti-H/K ATPase IgG autoantibodies were higher in females than males (*p* = 0.005) (Figure 7A). Finally, IgM autoantibodies directed against IFNγ were enriched in pSD males as compared to females (Figure 7A).

Next, we compared ANA-specific IgM and IgG autoreactivity in sera derived from NOD.B10, NOD.B10^*Tlr7+/−*^ and NOD.B10^*Tlr7−/−*^ females, and we observed no differences across the groups (Figures 7B, C). Finally, we assessed ANAs in NOD.B10 and NOD.B10^*Tlr7-/y*^ males. While we found no differences in ANA-specific IgG between the two groups, ANA-specific IgM was decreased in NOD.B10 *Tlr7*-deficient males as compared to their *Tlr7*-sufficient counterparts (Figures 7B, C). Indeed, IgM autoantibodies directed against CENP-A (*p* = 0.04), nucleosome (*p* = 0.04), Pm/Scl-100 (*p* = 0.04), SmD (*p* = 0.04), SP100 (*p* = 0.04), U1-snRNP-A (*p* = 0.04), nucleolin (*p* = 0.05), SRP54 (*p* = 0.05), Ku (p70/p80) (*p* = 0.07), PL-7 (*p* = 0.07), PL-12 (*p* = 0.07), Ro/SSA (52 kDa) (*p* = 0.08) and Pm/Scl-100 (*p* = 0.09) were diminished in NOD.B10 males that lacked *Tlr7* expression (Figure 7B).

### Expression of Tlr7 and Tlr9 is altered in pSD females as compared to males

**FIGURE 8:**
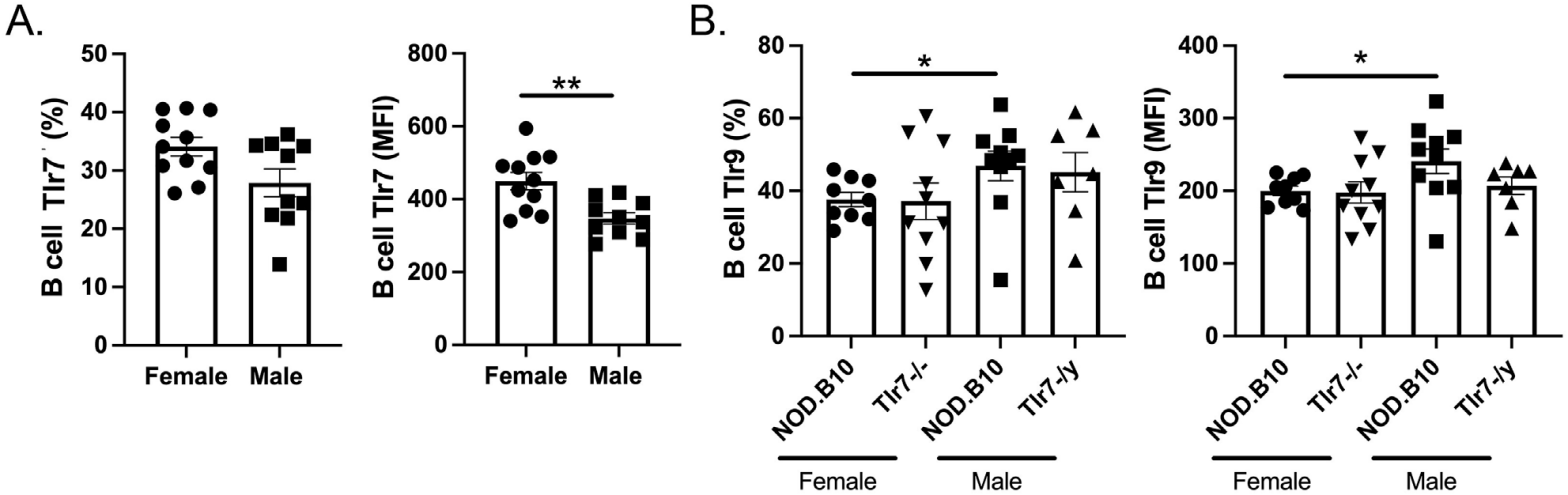
Tlr7 and Tlr9 expression levels are altered in pSD females as compared to males. Spleens were harvested from NOD.B10 female and male mice. Flow cytometry was performed to quantify the **(A)** percentage of Tlr7-expressing B cells and B cell Tlr7 MFI in NOD.B10 females (n = 11) and males (n = 9) and **(B)** the percentage of Tlr9-expressing B cells and Tlr9 MFI in NOD.B10 (n = 10) and NOD.B10^*Tlr7−/−*^ females (n = 10) and NOD.B10 (n = 10) and NOD.B10^*Tlr7y/-*^ males (n = 7). Horizontal lines represent mean and SEM, (*p < 0.05, **p > 0.01 for the hypothesis tests described in the statistical section).

**FIGURE 9:**
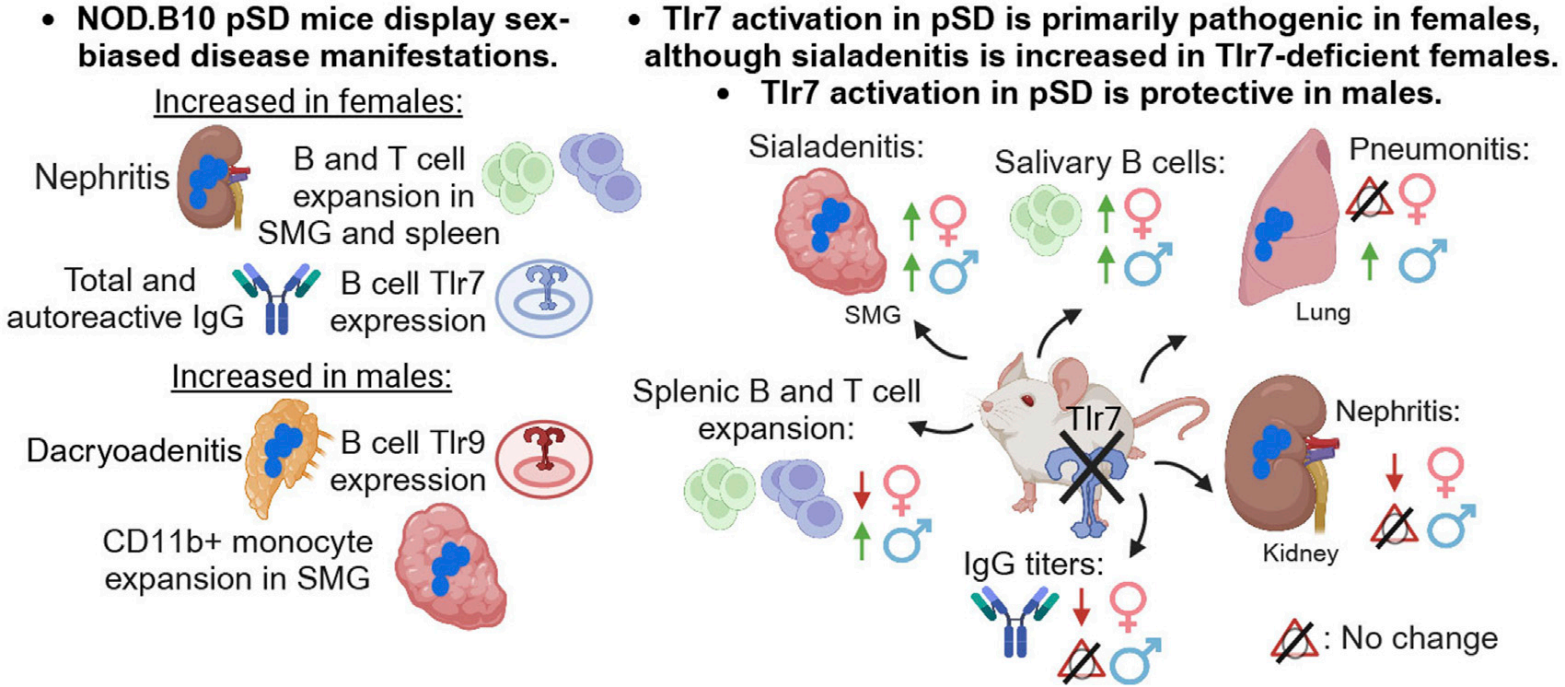
Summary. A summary of the major findings in both females and males and in *Tlr7*-deficient mice of both sexes is provided. This figure was created using BioRender software.

Lastly, we sought to determine if differences in the expression of Tlr7 and Tlr9 could contribute to the seemingly opposing role of Tlr7 in males and females. We focused our analyses on the B cell compartment, as B cell-intrinsic Tlr7 mediates lupus pathogenesis (Satterthwaite, 2021) and overexpression of Tlr9 in B cells protects against lupus (Tilstra et al., 2020). Although we found no difference in the percentage of B cells that expressed Tlr7 between males and females (Figure 8A), we observed that female mice had greater expression of B cell Tlr7 as measured by MFI (*p* = 0.004) (Figure 8A). Since Tlr9 activation diminishes Tlr7-mediated pathology in the context of lupus (Cosgrove et al., 2023), we next performed flow cytometry to assess expression of Tlr9 in *Tlr7*-deficient males and females. Our data revealed that pSD males express higher levels of Tlr9 as compared to females, both in terms of the percentage of B cells that express Tlr9 (*p* = 0.01) and MFI (*p* = 0.05) (Figure 8B). In the absence of Tlr7, Tlr9 levels are similar between NOD.B10 mice and their sex-matched Tlr7-deficient counterparts (Figure 8B). Thus NOD.B10 females express higher levels of Tlr7 and lower levels of Tlr9 in splenic B cells as compared to males. A summary of the major findings in male and female pSD mice and in sex-matched *Tlr7*-deficient animals presented herein is provided in Figure 9.

## Discussion

Recent clinical reports show that pSD patients exhibit sex-biased disease (Park et al., 2020; Ramirez Sepulveda et al., 2017; Bruno et al., 2022; Liu et al., 2024). Findings from the present study demonstrate that Tlr7 drives dichotomous disease manifestations in a sex-specific manner in the context of pSD. To begin to assess the molecular mechanisms that underlie the clinical differences observed between the sexes, we performed studies to examine distinct disease phenotypes in pSD males and females in *Tlr7*-deficient mice. Our data revealed that Tlr7 signaling is primarily pathogenic in females, as evidenced by decreased nephritis, diminished B and T cell populations associated with disease, and reduced serum IgG titers in *Tlr7*-deficient pSD females. In contrast, Tlr7 expression was primarily protective in male pSD mice, as ablation of *Tlr7* in males resulted in elevated sialadenitis and pneumonitis along with increased B and T cell populations as compared to Tlr7-sufficient pSD males. Altogether, these data demonstrate that Tlr7 contributes to pSD pathogenesis in a tissue-specific and sex-biased manner, and mediates distinct and often opposing disease manifestations in males and females.

It is important to point out that TLR7 signaling and expression is sex-dependent in humans, as there is good evidence that TLR7 escapes XCI in immune cells in both health and disease, leading to higher expression in females as compared to males. Indeed, TLR7 escapes XCI in monocytes, B cells, and pDCs in healthy women (Souyris et al., 2018; Hagen et al., 2020). TLR7 expression is reported to be elevated in both minor salivary glands and parotid tissue from pSD patients, as well as B cells, pDCs, CD14^+^ monocytes and monocytic DCs (Punnanitinont and Kramer, 2023). Most of these studies, however, were conducted on patient populations that were primarily female, so further work is needed to determine whether TLR7 expression is increased in specific cells and tissues in female pSD patients as compared to males.

Our study revealed that Tlr7 exhibits differential, organ-specific manifestations in pSD males and females. While the reasons for these findings are incompletely understood, the underlying mechanisms that govern these observations are complex and likely include differential expression of mediators of TLR7 signaling, such as TLR9. This has been studied most extensively in B cells, where TLR9 expression in autoreactive B cells constrains BCR/TLR7-mediated signaling (Cosgrove et al., 2023; Nundel et al., 2015). Studies in our lab are ongoing to determine if regulation of TLR7 by TLR9 mediates organ-specific manifestations of pSD. TLR7 expression and function is governed by several mechanisms in addition to TLR9. Specifically, type I IFN signaling controls TLR7 levels and UNC93B1 is also a key regulator of TLR7 signaling (Severa et al., 2007; Mohty et al., 2003; Majer et al., 2019; Fukui et al., 2011; Rael et al., 2024; Wolf et al., 2024). Alterations in IFN or UNC93B1 levels could results in differential TLR7 expression in a tissue-specific manner (Nakano et al., 2010), although whether this occurs in pSD remains to be determined. Finally, TLR7 activation by endogenous ligands could vary in different organs. For example, since TLR7 can be activated by apoptotic debris (Vinuesa et al., 2023), and studies suggest that efferocytosis occurs improperly in pSD (Manoussakis et al., 2014; Witas et al., 2021; Witas et al., 2022), it is possible that release of self-derived TLR7 agonists may be enhanced in particular tissues that are damaged in disease, and this could lead to heightened TLR7 activation in distinct organs. Additional work is needed to determine whether these putative mechanisms drive TLR7-dependent pSD organ-specific manifestations.

While this is the first study, to our knowledge, examining the role of Tlr7 signaling in a pSD model, two studies have examined the role of Tlr7 in secondary SD models. Similar to our findings, one study in NOD/ShiLtJ mice that develop SD and type I diabetes (TID) simultaneously found that Tlr7 was not required for salivary gland inflammation in females [(Debreceni et al., 2020) and Figure 2C]. In contrast to results reported herein (Figures 2D, E), the study in NOD/ShiLtJ animals also found that ablation of *Tlr7* protected males from lacrimal gland inflammation (Debreceni et al., 2020). It is important to point out that ablation of Tlr7 in the NOD/ShiLtJ model also protected male mice from development of TID (Debreceni et al., 2020). This is significant because hyperglycemia seen in the context of TID in this strain mediates inflammatory cytokine production in salivary tissue (Allushi et al., 2019). Thus, it is possible that *Tlr7*-deficient NOD/ShiLtJ male animals had reduced hyperglycemia as compared *Tlr7*-sufficient controls, and this may have also indirectly diminished the inflammation within the lacrimal tissue. This reduced TID severity could account for the disparate findings observed between the present study and the prior report (Debreceni et al., 2020), although further work is needed to establish this conclusively.

A second study examined the effects of *Tlr7* ablation in *Tlr8* ^*−/−*^ female mice that develop both lupus and SD-related autoimmunity contemporaneously (Wang et al., 2021; Demaria et al., 2010). In contrast to our findings, data from this study showed that both salivary and lung inflammation were diminished in animals that lacked both *Tlr7* and *Tlr8* expression as compared to *Tlr8* ^*−/−*^ controls (Wang et al., 2021). In addition, autoantibodies that are common to both lupus and SD, specifically anti-SSA (Ro), anti-SSB (La), and anti-RNA, were diminished in the double-knockout mice as compared to *Tlr8*-deficient animals (Wang et al., 2021). These findings are also distinct from those reported in the current study, as ablation of *Tlr7* in female NOD.B10 mice resulted in enhanced sialadenitis and did not alter the degree of inflammation in the lungs (Figure 2B and Figure 4E, respectively). Moreover, we saw no differences in autoantibodies between NOD.B10 and NOD.B10^*Tlr7−/−*^ females (Figures 6C, 7B). It is likely that these opposing results are due to the fact that the *Tlr8* ^*−/−*^ mice also displayed a lupus-like disease, and the presence of this additional underlying disease likely altered autoantibody titers as well as salivary and lung inflammation in these mice. These results highlight the importance of conducting studies in pSD models to determine the way in which Tlr7 expression governs pathology in the primary form of the disease specifically.

While ablation of Tlr7 in NOD.B10 females diminished total IgG in the sera, it did not alter autoantibody levels. This finding was surprising, as Tlr7 is required for expression of RNA-associated autoantibodies in lupus (Nickerson et al., 2010). Of note, prior work in our lab revealed that treatment of pre-disease female NOD.B10 mice with Imq accelerated disease and mediated increased expression of RNA-associated autoantigens in the sera of NOD.B10 mice as compared to Imq-treated C57BL/10 controls (Punnanitinont et al., 2022). In the current study, however, ablation of *Tlr7* had no effect on the production of these autoantibodies in female NOD.B10 mice, suggesting that endogenous activation of B cells by classical RNA-associated antigens may be negligible in our model, at least at the clinical disease stage. Table 1 provides a comprehensive overview of findings in Imq-treated NOD.B10 females as compared to those in Tlr7-deficient females and males.

**TABLE 1:** Phenotypic features of Imq-treated and *Tlr7*-deficient pSD strains.

Disease feature	Imq-treated (female)^a^	*Tlr7* ^*−/−*^(Female)^b^	*Tlr7-/y* (Male)^b^
Sialadenitis	Increased	Increased	Increased
Saliva production	Decreased	N.D.	N.D.
Dacroyadenitis	Increased	N.D.	N.D.
Nephritis	Increased	Decreased	N.D.
Pneumonitis	Increased	N.D.	Increased
Total IgM	N.D.	N.D.	N.D.
Total IgG	Increased	Decreased	N.D.
IgM-specific ANAs	Increased	N.D.	Decreased
IgG-specific ANAs	Increased	N.D.	N.D.
T-bet + ABCs (spl)	Increased	N.D.	Increased
T-bet+ CD11c+ ABCs (spl)	N.D.	Decreased	N.D.
CD4^+^ (Act/Mem) (spl)	N.D.	N.D.	Increased
CD4^+^ (Act/Mem) (cLN)	Increased	N.D.	N.D.

a Compared to NOD.B10 sham-treated controls.

b Compared to age- and sex-matched NOD.B10 mice, N.D., No difference; Spl, spleen.

It is important to point out that while our prior study revealed that treatment of NOD.B10 females at the pre-disease stage with Imq resulted in heightened salivary inflammation and loss of salivary flow. In the current study, we found that males and females that lack Tlr7 expression also have increased sialadenitis in salivary tissue and similar salivary production as compared to the parental strain (Table 1). While these 2 findings seem disparate at first, there are several possible explanations as to why both ablation and agonism of Tlr7 may lead to heightened inflammation in the salivary tissue. TLR7 signals as a homodimer with two ligand binding sites, “site 1” and “site 2” (Zhang et al., 2018). Each site has different affinities for endogenous and exogenous ligands; this differential binding capability alters downstream signaling cascades (Zhang et al., 2018). In the prior study from our group (Punnanitinont et al., 2022), mice were treated with Imq, which mimics exogenous (viral) activation (Hemmi et al., 2002), while in the current study, we examined the animals in a more physiologic context whereby loss of endogenous Tlr7-mediated signals enhanced salivary inflammation (Figures 2, 3).

Since there is considerable cross-regulation between pattern recognition receptors (PRRs) (Tan et al., 2014), it is likely that loss of Tlr7 signaling enables heightened activation of other pathways that control inflammation. Indeed, Tlr7 is a Myd88-dependent TLR and Myd88 is an inhibitor of Trif signaling. In corneal epithelial cells, loss of Myd88 caused increased Tlr3/Trif-dependent RANTES expression (Johnson et al., 2008). Consistent with this report, we have observed that ablation of Myd88 in either immune cells or in the stromal compartment results in production of both pro- and anti-inflammatory mediators in SMG tissue derived from NOD.B10 females (Kiripolsky et al., 2021). Another possibility to explain the differential effects of Tlr7 in SMG tissue is that loss of Tlr7 expression may mediate increased expression of endogenous retroviruses (ERVs) in salivary tissue, which then elicit tissue-specific inflammation. Indeed, elegant studies in healthy mice demonstrate that Tlr7 is the most important endosomal Tlr for the suppression of endogenous antiretroviral immune responses (Yu et al., 2012). Moreover, lymphoid tissue from *Tlr7* ^*−/−*^ mice has strong ERV mRNA expression (Yu et al., 2012). These data suggest that in salivary tissue, the absence of Tlr7 could enable enhanced ERV-driven inflammation through activation of other PRRs, although further studies are needed to determine the underlying mechanisms that mediate the heightened inflammation observed.

There is one recent study, to our knowledge, that demonstrates an essential role for salivary gland inflammation mediated by Tlr7 in pSD-like disease. Indeed, Lysosome-Associated Membrane Protein 3 (LAMP3) is expressed in salivary epithelial cells, and LAMP3 expression is elevated in salivary tissue from pSD patients as compared to controls (Nakamura et al., 2024). When LAMP3 was overexpressed in salivary tissue of healthy female mice, the animals developed a heightened type I IFN gene signature and elevated Tlr7 expression in the salivary epithelium (Nakamura et al., 2024). Moreover, these mice develop robust salivary inflammation, loss of salivary flow, and elevated anti-Ro autoantibodies in sera (Nakamura et al., 2024). These disease manifestations were dependent on Tlr7, because when LAMP3 was overexpressed in healthy *Tlr7*-deficient mice, the mice were protected from local and systemic disease (Nakamura et al., 2024). Thus, this study provides compelling evidence that Tlr7 activation in salivary tissue contributes to pSD.

It is interesting to note that several IgM-specific ANAs were reduced in NOD.B10 *Tlr7*-deficient males as compared to their *Tlr7*-sufficient counterparts, including those that contain RNA and activate Tlr7 such as Ro/SSA (60 kDa), Sm, SmD, and U1-SnRNP-A (Figure 6B) (Pisitkun et al., 2006; Deane et al., 2007), although this self-reactivity was not observed in the IgG compartment. Autoreactive IgM is associated with autoimmunity, but the role of these autoantibodies in disease is controversial. Some studies suggest that elevated IgM production is part of a compensatory protective response rather than a driver of disease, as high levels of anti-dsDNA IgM autoantibodies in SLE patients are negatively correlated with renal disease (Nguyen and Baumgarth, 2016). Since *Tlr7* ablation was primarily protective in male pSD mice, and ANA-specific IgM autoantibodies were reduced in *Tlr7*-deficient males while IgG autoantibodies remained unchanged (Figures 7B, C), it is possible that these IgM autoantibodies are pathogenic in the context of pSD. Indeed, elevated IgM antibodies are associated with distinct disease manifestations in pSD patients (Zhao et al., 2015). Thus, further studies are needed to determine whether autoreactive IgM is pathogenic or protective in the context of pSD.

Of direct relevance to the current work, a recent retrospective study compared clinical disease in male (n = 37) and female pSD patients (n = 367) in a Chinese population (Liu et al., 2024). Importantly, male patients displayed several key differences in clinical disease, serology, and in peripheral blood cell populations (Liu et al., 2024). Similarities between their findings and those reported herein were noted, as xeropthalmia was greater in men than women (Liu et al., 2024), which may be consistent with the robust lacrimal inflammation in NOD.B10 males as compared to females (Figure 2). Moreover, similarities in peripheral blood findings were observed, as pSD male mice and humans displayed decreased percentages of CD4^+^ T cells as compared to females, with no changes in CD8^+^ T cell or B cell percentages [(Liu et al., 2024) and Figure 5]. Of note, male pSD patients were significantly older than females at the time of diagnosis, and displayed higher IgG levels in sera and more robust pulmonary and renal pathology (Liu et al., 2024). While we did not note these alterations in our study, it is important to point out that all of our animals were assayed at the same age, so additional studies are needed in aged mice to determine whether NOD.B10 males develop disease manifestations that are reminiscent of those observed in the Chinese cohort (Liu et al., 2024).

While the reasons that underly the sex-biased role of Tlr7 in pSD are unknown, studies in the lupus field demonstrate that Tlr9 plays a key role in regulating Tlr7-driven immune activation, as *Tlr9* ^*−/−*^ lupus mice show exacerbated disease that is ameliorated when Tlr7 is also deleted (Nickerson et al., 2010). More recent work revealed that in lupus mice that lacked *Tlr9* systemically, conditional ablation of *Tlr7* in B cells ameliorated disease (Cosgrove et al., 2023). Thus, expression of Tlr7 in the B cell compartment was of critical importance in mediating disease driven by the lack of Tlr9 (Cosgrove et al., 2023). In the current study, we found that B cell Tlr7 levels are decreased in males as compared to females, and an inverse relationship is observed regarding Tlr9 expression (Figure 8). These data suggest that the attenuated disease observed in male pSD mice could be mediated by the relatively low Tlr7 expression coupled with higher Tlr9 levels in B cells. Indeed, overexpression of B cell Tlr9 in a lupus model protected against lupus nephritis (Tilstra et al., 2020). While it is unclear if this paradigm extends to pSD, further studies in pSD mice lacking *Tlr9* alone and in animals deficient in *Tlr7* and *Tlr9* in combination will be insightful.

In conclusion, these data demonstrate that NOD.B10 mice display sex-biased disease that is reminiscent of pSD patients. This sexual dimorphism may be mediated, at least in part, by differential expression of Tlr7 and Tlr9 in the B cell compartment of males and females. *Tlr7*-deficient females were protected from pSD, while ablation of *Tlr7* in males exacerbated disease. Thus, Tlr7 is a potent mediator of pSD in both sexes, and therapeutics that target these pathways will likely be efficacious in mitigating sex- and tissue-specific disease manifestations.

## Data Availability

The datasets presented in this study can be found in online repositories. The names of the repository/repositories and accession number(s) can be found below: https://www.ncbi.nlm.nih.gov/geo/, GSE255215.
