# Supplementary material for: Tlr7 drives sex- and tissue-dependent effects in Sjögren’s disease
Source: Front Cell Dev Biol. 2024 Sep 6;12:1434269. doi: 10.3389/fcell.2024.1434269 (PMC11413591; doi:10.3389/fcell.2024.1434269)
Supplement: Supplementary file 1 [file DataSheet2.PDF]

Supplemental Figure 2

Flow cytometry gating stategies - Spleen and cLNs

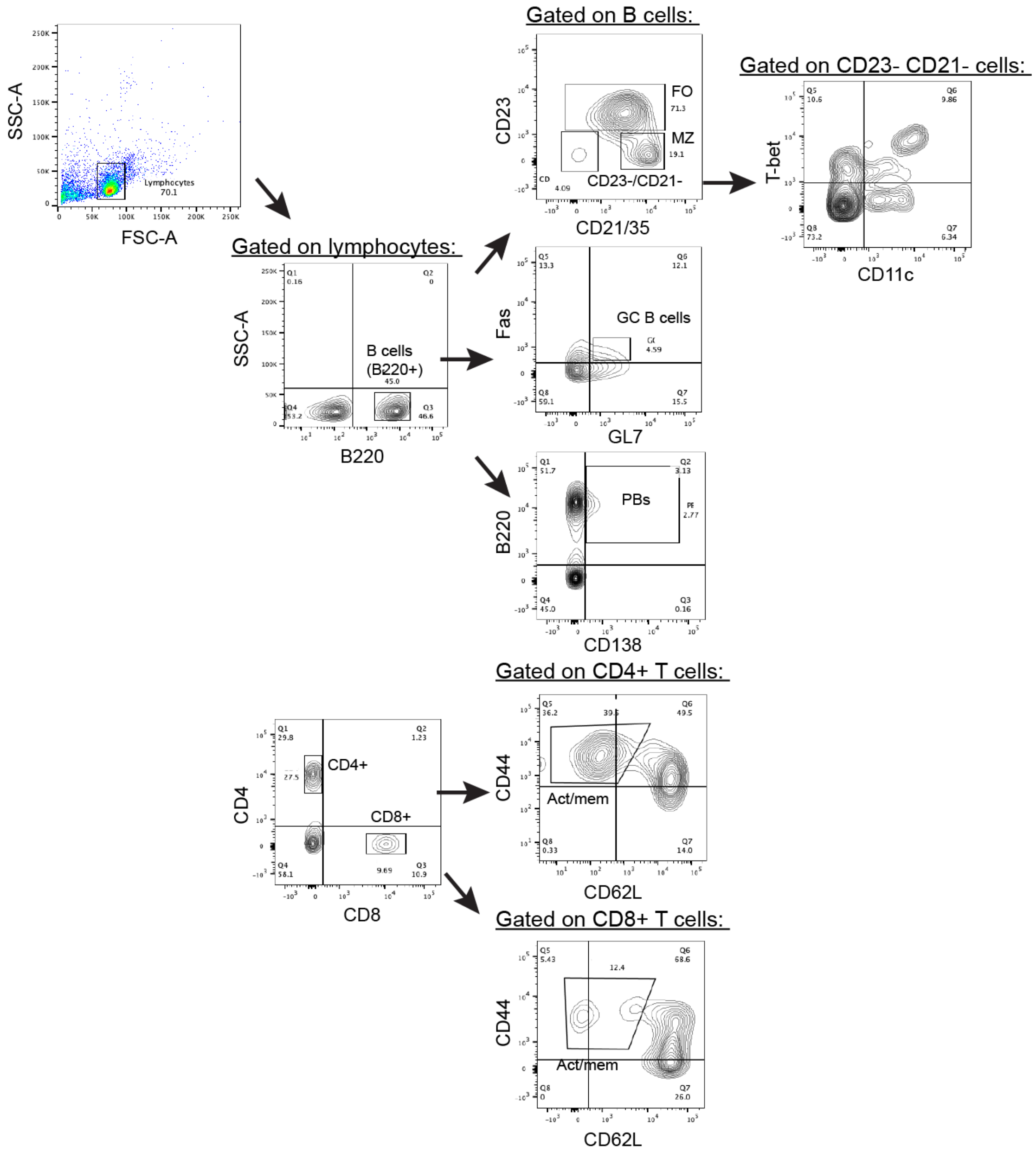

Supplemental Figure 2: Flow cytometry gating strategy. The gating strategy for splenocytes from a representative female NOD.B10 mouse is shown. Abbreviations: FO = Follicular, MZ = Marginal Zone, GC = Germinal Center, PBs = Plasmablasts, Act/mem = Activated /memory.
