# Supplementary material for: Tlr7 drives sex- and tissue-dependent effects in Sjögren’s disease
Source: Front Cell Dev Biol. 2024 Sep 6;12:1434269. doi: 10.3389/fcell.2024.1434269 (PMC11413591; doi:10.3389/fcell.2024.1434269)
Supplement: Supplementary file 2 [file DataSheet1.PDF]

Supplemental Figure 1

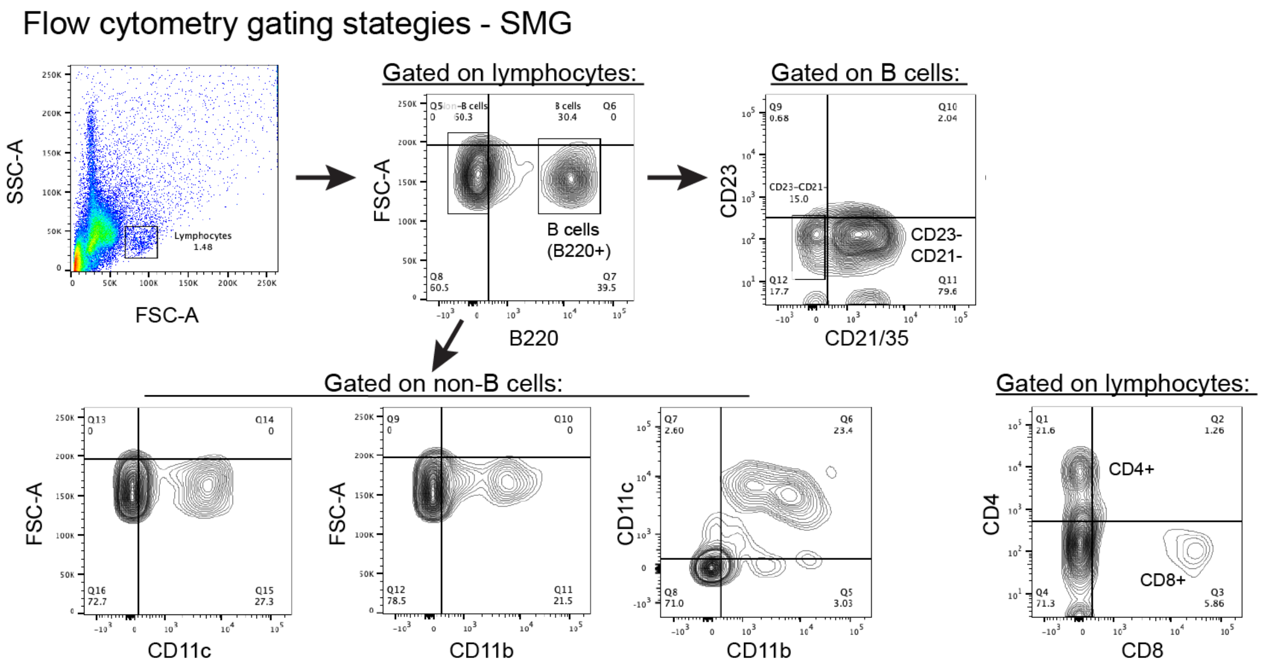

Supplemental Figure 1: Flow cytometry gating strategy. The gating strategy for SMGs from a representative female NOD.B10 mouse is shown.
